# Perception and Impact of Long-Term Oxygen Therapy on the Functioning and Quality of Life of Patients With Chronic Respiratory Disease: A Mixed-Method Study

**DOI:** 10.7759/cureus.63091

**Published:** 2024-06-25

**Authors:** Mariya P Jiandani, Khushali B Jain, Pramila K Lohakare

**Affiliations:** 1 Department of Cardiovascular and Respiratory Physiotherapy, Physiotherapy School and Center, Seth Gordhandas Sunderdas Medical College (GSMC) and King Edward Memorial (KEM) Hospital, Mumbai, IND

**Keywords:** long-term oxygen therapy, functioning, chronic respiratory diseases, activities of daily living, quality of life, perception

## Abstract

Introduction

In patients with severe chronic pulmonary diseases, there is often a need for oxygen therapy to continue after discharge from hospitalization. Long-term oxygen therapy (LTOT) has been shown to significantly reduce mortality in such patients and improve longevity by helping to correct oxygen deficiency in the bloodstream and prevent organ failure and the development of cor pulmonale (right-sided heart failure). Therefore, considering the sociocultural background of India, the objective of the present study was to evaluate patients’ perceptions of LTOT using semi-structured interviews, to evaluate patients’ perceptions of activities and participation, and to evaluate the quality of life (QOL) of patients with LTOT.

Methodology

A mixed-method study was performed at a tertiary care hospital for six months. Twenty-four chronic respiratory patients were included in the present study. The patients' perception was evaluated about LTOT using semi-structured interviews, activities, and participation using a validated activity and participation checklist and the QOL of patients with LTOT using the World Health Organization Quality of Life Brief Version (WHOQOL-BREF) questionnaire.

Results

Twenty-four patients were interviewed and transcripts were analyzed through thematic analysis. Descriptive statistical analysis was performed for activity and participation along with QOL. The mean age of the patients involved was 58.5 ± 9.54 years, which involved a maximum of male patients consisting of 13 (54.2%) in comparison to female patients. The duration of oxygen use in months was 31.4 ± 29.4, the daily oxygen usage in hours was 17.3 ± 6.6, and the oxygen flow rate (L/min) was found to be 2.3 ± 0.97 at rest and 3.6 ± 1.4 on activity. In addition, the oxygen use by the patients was preferable as prescribed by 15 (62.5%) patients. Patients' perspectives on LTOT demonstrated that 10 (41.7%) patients perceived oxygen as relieving symptoms while most patients used oxygen during walking indoors activity involving 22 patients (91.7%), with 17 (77.3%) reporting improved ability and five (22.7%) facing obstacles. Instrumental activities involving walking shorter distances (less than 1 km) involved a high usage of oxygen with 20 patients (83.3%) using it, where 15 (75%) found it beneficial, three (15%) encountered obstacles, and two (10%) noted no effect from its use. Social interaction found that only one patient (4.20%) used oxygen at work, finding it helpful, but the majority, 20 (83.4%), did not go to work at all. Moreover, oxygen usage during transportation reported that travel using private vehicles involved a maximum of patients (16, 66.7%). Furthermore, for inquiries related to QOL, the results demonstrated that for the four domains of WHOQOL-BREF, consisting of physical health, psychological, social relationships, and environment, the mean values were found to be 48.33 ±10.66, 54.79 ± 13.7, 55.75 ±11.1, and 60.25 ± 12.6, respectively.

Conclusion

LTOT has been perceived to be a life-saving intervention by majority of the chronic respiratory disease patients of increased severity. Patients experienced various issues in daily activities and participation, which have affected their QOL. Overall, a lack of awareness and knowledge regarding the purpose, dosage, benefit, and usage of oxygen therapy was found to be evident and needs to be focused.

## Introduction

Chronic lower respiratory disorders evidence to rank seventh in terms of morbidity burden and fourth in terms of the global cause of mortality by 2030 [1]. India has 18% of the world’s population and consists of 32% of the global burden of respiratory diseases, which is second to that of ischemic heart diseases, as reported by a state-level analysis of the Global Burden of Disease data. The corresponding figures for 1990 were 9.6% and 4.5%, respectively [2,3]. In patients with severe chronic pulmonary diseases, there is often a need for oxygen therapy to continue after discharge from hospitalization [1]. The major landmark in this area were the two randomized controlled clinical trials, The Nocturnal Oxygen Therapy Trial (NOTT 1980) [4] and the Medical Research Council (MRC 1981) [5] study, which showed that long-term oxygen therapy (LTOT) was the sole treatment that improved survival in patients with chronic obstructive pulmonary diseases (COPD) and chronic respiratory failure, which was reaffirmed by Dubois et al. (1994) [6] and Zielinski et al. (1998) [7]. To achieve clinical outcomes, LTOT should be utilized for 15 hours each day [8,9].

LTOT has been shown to significantly reduce mortality in such patients and improve longevity by helping to correct oxygen deficiency in the bloodstream and prevent organ failure and the development of cor pulmonale (right-sided heart failure) [10]. Numerous research studies have demonstrated additional advantages, including the stabilization of pulmonary arterial hypertension, a decrease in cardiac arrhythmias, a rise in exercise tolerance, a decrease in hospitalizations or exacerbations, and an enhancement of neuropsychiatric functions, like memory, intellect, motor skills, abstraction, and perceptual motor ability [11,12]. For use at home, a variety of equipment is available, such as cylinders, portable systems, liquid systems, and concentrators. The patient's clinical status, the required inspired oxygen concentration, and the patient's tolerance to the device all play a role in the oxygen delivery system selection. Although the benefits of LTOT, such as reduced mortality and improved exercise capacity, are well-documented, much of the existing research is derived from the studies conducted in Western countries with healthcare systems and patient demographics that significantly differ from those in developing countries like India.

Moreover, several studies have been documented in Western literature trying to understand the perceptions of patients of COPD with oxygen therapy. They have focused on patients’ perceptions regarding the benefits and limitations imposed by oxygen therapy and its impact on their functional independence, such as the patterns of adherence to supplemental oxygen, such as full-time use, needed use, and part-time use in people with hypoxemic COPD. Adherence is a challenging task with numerous obstacles, such as the inability to use oxygen due to physical difficulties, social stigma and self-consciousness, a lack of perceived benefit, difficulties performing activities of daily living (ADLs), and fear of negative treatment side effects. Many different and complicated social and educational aspects affect oxygen therapy [8].

India, as a developing country, has a very different cultural background, belief patterns, adaptations, and support systems. The sociocultural dimensions influencing the adoption and efficacy of LTOT, such as patient perceptions, stigma associated with oxygen use, and local healthcare practices, are underexplored in the Indian context. Qualitative research on LTOT administration and patient perception may throw light on new evidence regarding emotional, physical, psychological, cultural, and social functions [13]. Given that cultural beliefs and social support systems play a crucial role in the management of chronic diseases, there is a pressing need for comprehensive studies that address these factors. Hence, this study aimed to fill the gap by examining the perceptions and impacts of LTOT on daily functioning and quality of life (QOL), among patients in India, thereby offering insights that could inform culturally sensitive clinical practices and aid in policy making.

## Materials and methods

After approval from the Institutional Ethical Committee (reference number EC/193/2019) and clinical trial registration (reference number CTRI/2020/11/028910), a mixed-method study was performed at Physiotherapy School and Center, Seth Gordhandas Sunderdas Medical College (GSMC) and King Edward Memorial (KEM) Hospital, a tertiary care hospital in Mumbai, India, for six months. Patients with chronic respiratory disorders of either a documented diagnosis of COPD or interstitial lung disease (ILD)-prescribed oxygen therapy for more than six months, and patients who were able to comprehend in either of the languages involving English, Marathi, and Hindi were included in the present study. Meanwhile, patients with additional comorbidities of heart failure that would affect oxygen usage, patients with a locomotor disability that would affect ambulation, patients with cognitive or language difficulties, patients who have been recently hospitalized that is within the last three months, and patients not willing to participate were excluded from the present study.

Through the complete enumeration method, a total of 24 patients were enrolled up to the point of data saturation for which three to four patients per month were considered. A written informed consent was taken from the recruited patients, and the data collection period lasted six months. The outcome measures evaluated consisted of themes arising from the semi-structured interviews, validated activity, and participation checklist for patients with LTOT to know their perceptions about LTOT and their activity and participation in society and QOL with the World Health Organization Quality of Life Brief Version (WHOQOL-BREF) questionnaire (see Appendix).

For the preparation of the questions for the semi-structured interviews, a literature search was carried out on Google Scholar and PubMed databases using keywords such as "long-term oxygen therapy," "perception," "oxygen therapy," and "patient’s perceptions." After reviewing the literature, major domains of the perception regarding LTOT were identified. Considering the domains, 20 questions were formulated for the interview, out of which seven were removed as they were not relevant to the objective of the present study. The questions enquired about beliefs regarding oxygen therapy, the mobility status of the patients, potential benefits, problems with oxygen therapy, ability to perform exercise, thoughts about the role of exercise, and their experiences with oxygen in the social environment. Two independent subject experts consisting of a physiotherapist and a psychiatrist reviewed the questions to verify that the objective was fulfilled to its entire potential. Based on the review, the two experts suggested an addition of one more question regarding the patient’s worries about living with oxygen therapy, summing the total number of questions to 14. These semi-structured questions were validated by five subject experts and by two patients.

For the preparation of the checklist, a list of items representing activities and participation was generated using the International Classification of Function (ICF) and the Barthel index. The list of items included ADLs, such as eating, bathing, toileting, instrumental activities involving laundry, cooking food, participation in society, and the use of transport. Subsequently, a checklist was prepared consisting of 25 items that gave a broad idea regarding the usage of oxygen during various activities and participations in society, which was validated by subject expert professionals and seven patients. Their responses were then formulated into a spreadsheet, and the checklist was finalized after making appropriate changes. It was then translated into Hindi and Marathi languages and was validated after forward and backward translations.

For commencing the interview, the patients were connected through telephone, and a suitable time was fixed for the interview. All the patients underwent a telephonic interview except one where a face-to-face interview was conducted as the patient had come for follow-up in the outpatient department (OPD) and as per the patient’s request. The informed consent of all the participants was audio-recorded, and the purpose and nature of the study were explained to the patients in their best-known language. Prior to the interview, the patients were assured that if they faced any discomfort, they could take a pause or it could be rescheduled at their convenience. The demographic data and the information related to the disease along with oxygen therapy were noted. The QOL was measured using the WHOQOL-BREF questionnaire, which contains a total of 26 questions under four domains that involved physical health (seven items), psychological health (six items), social relationships (four items), and environment (nine items) that reflect an individual’s perception of QOL in each domain. Every single question in the WHOQOL-BREF has a response scale, which is a five-point ordinal scale, with scores ranging from 1 to 5. After that, the scores are linearly converted to a 0-100 scale [14]. The patients' responses to the WHOQOL-BREF questionnaire and checklist for activity and participation in local languages were noted. The semi-structured interview was conducted in the local languages as understood by the patient, and the interviews were audio-recorded with the patients' permission. The whole session lasted for approximately 40 to 50 minutes. For around eight patients, the interview session had to be conducted in two parts as the patients were not comfortable talking for longer periods.

The interviews were transcribed and anonymized, and the transcripts were corrected for any discrepancies and were confirmed by the patients through subsequent validation interviews. The transcripts were analyzed initially using open coding to identify data relevant to the objective of the study. The relevant data were grouped into broad codes or headings. The similarities and differences between these codes were compared by analyzing the transcripts through constant comparisons and links between categories and emerging themes were confirmed. To verify the analysis process, a second author separately reviewed the transcripts and produced the codes for a few of the sample patients. There was no contradiction between both analyses. However, it suggested areas requiring additional coding and emphasized the data that might have been ignored and also the supporting data. The resultant themes were formed that could best reflect the patient’s perception regarding LTOT. The responses from the activity and participation checklist and from the transcripts were compared and analyzed further to develop a comprehensive understanding of the patient’s activity status with LTOT through the triangulation method. Data from the WHOQOL-BREF were analyzed using descriptive statistics. Data were analyzed and reported in terms of mean and standard deviations and frequency and percentages.

## Results

This study incorporated a mixed-method approach to know the perception of patients on LTOT and their activities and participation in society along with the QOL. Twenty-four participants were interviewed, and the transcripts were analyzed through thematic analysis. The patients' mean age was 58.5 ± 9.54 years (ranging from 33 to 77 years). In addition, male patients were 13 (54.2%), higher than the number of female patients (11, 45.8%). 

Oxygen therapy of the patients

The descriptive statistics about the oxygen therapy of the patients are demonstrated in Table 1.

**Table 1:** Descriptive statistics related to the oxygen therapy of the patients SD: standard deviation

Characteristics		Mean ± SD	Range
Duration of oxygen use in months		31.4 ± 29.4	6 to 120
Daily oxygen usage in hours		17.3 ± 6.6	8 to 24
Oxygen flow rate (L/min)	At rest	2.3 ± 0.97	1 to 5
	On activity	3.6 ± 1.4	2 to 8

Table 2 demonstrates the oxygen use patterns and equipment type.

**Table 2:** Oxygen use patterns and equipment type

Oxygen use patterns and equipment type in frequency (n) and percentage (%)		
Oxygen use values reported	Full time	11 (45.8%)
	Part time	13 (54.2%)
	As prescribed	15 (62.5%)
	Self-regulated	9 (37.5%)
Type of equipment used by 24 participants values reported	Concentrator	24 (100%)
	Backup cylinder	11 (45.8%)
	Portable	8 (33.3%)
	All	5 (20.8%)
Use of portable by eight participants values reported	Outside the house	6 (75%)
	Inside the house	2 (25%)
Rental oxygen equipment values reported	Yes	11 (45.8%)
	No	13 (54.2%)
Financial help values reported	Yes	3 (12.5%)
	No	21 (87.5%)

Patients' perception on LTOT

Patients' perspectives on LTOT vary widely, with 10 (41.7%) patients perceiving oxygen as relieving symptoms and 14 (58.3%) patients denying this perception. Although none viewed oxygen as inherently harmful, notably, seven (29.2%) were uncertain about its potential negative impacts. The majority of the patients (17, 70.8%) reported that oxygen therapy affects their societal roles. Conversely, seven (29.2%) did not perceive any impact on their role in society. A large proportion of patients (18, 75%) expressed concerns about living with oxygen therapy. On the other hand, six (25%) did not share these concerns. About 16 (66.7%) of the patients felt that they were sufficiently informed about the oxygen equipment they use. However, eight (33.3%) felt that they lacked adequate information.

Impact of oxygen therapy on ADLs

Oxygen use varied significantly across different ADLs. For eating, 11 patients (45.8%) used oxygen, with the majority (10, 90.9%) reporting that it aided their ability to perform this activity, while one patient (9.1%) noticed no difference. The remaining 13 patients (54.2%) did not use oxygen while eating. During grooming, 14 patients (58.3%) used oxygen, and 12 (85.7%) found it beneficial; however, two patients (14.3%) encountered difficulties. Ten patients (41.7%) did not use oxygen for grooming, and three (12.5%) required additional assistance. Bathing showed similar trends, with 16 patients (66.7%) using oxygen. Of these, 12 (75%) reported benefits, whereas four (25%) faced challenges. Eight patients (33.3%) managed without oxygen, and five (20.8%) needed help. For toileting, 15 patients (62.5%) used oxygen, with 13 (86.7%) finding it helpful and two (13.3%) experiencing problems. Nine patients (37.5%) did not use oxygen for toileting, and three (12.5%) required assistance. Walking indoors was facilitated by oxygen for 22 (91.7%) patients, with 17 (77.3%) reporting improved ability and five (22.7%) facing obstacles. Only two patients (8.3%) walked without oxygen. Finally, for climbing stairs, half of the patients (12, 50%) used oxygen, all of whom found it helpful. The same number did not use oxygen or were unable to perform the activity, and six patients (25%) needed assistance.

Impact of oxygen therapy on instrumental ADLs

In a study of 24 patients, the use of oxygen therapy varied across several instrumental ADLs. For laundry, only four patients (16.7%) used oxygen, with the majority (20, 83.3%) not engaging in this activity at all. In the cooking activity, seven patients (29.2%) used oxygen and all found it beneficial. Fifteen patients (62.5%) did not cook, and seven (29.2%) required assistance while cooking. House cleaning saw similar levels of oxygen usage, with four patients (16.7%) using oxygen and finding it helpful, while 19 patients (79.2%) did not perform this task, and two (8.3%) needed assistance. Making the bed involved six patients (25%) who used oxygen, although 12 patients (50%) did not engage in this activity at all. When it came to shopping for groceries or vegetables, none of the 24 patients used oxygen for this task, and a large majority (18, 75%) did not perform this activity. Walking shorter distances (less than 1 km) involved a high usage of oxygen, with 20 patients (83.3%) using it, where 15 (75%) found it beneficial, three (15%) encountered obstacles, and two (10%) noted no effect from its use. For walking longer distances (more than 1 km), only six patients (25%) used oxygen, and all of them reported it to be helpful, while 15 patients (62.5%) did not engage in this activity, and five (20.8%) required assistance

Oxygen therapy and social participation

The impact of oxygen therapy on social participation varied across several activities. When going out of the home, eight (33.3%) used oxygen. About a quarter of the patients did not use oxygen for this activity, and a significant portion (10, 41.7%) avoided going out altogether, with nine (37.5%) requiring assistance. For social gatherings, a smaller group (4, 16.7%) used oxygen and all reported positive outcomes. However, a large majority (18, 75%) did not attend such events at all. Similarly, when visiting religious places, only five patients (20.8%) used oxygen, but most patients (16, 66.7%) refrained from participating in these activities. Engagement with children showed that 12 patients (50%) used oxygen. Leisure activities showed 11 patients (45.8%) using oxygen with positive feedback, although over half did not use it for such activities. Participation in exercise or rehabilitation programs was higher, with 16 patients (66.7%) using oxygen. Medical follow-up visits also had a high usage of oxygen in 15 patients (62.5%). Work-related activities and going out of town were less frequent. Only one patient (4.20%) used oxygen at work, but the majority (20, 83.4%) did not go to work at all. For out-of-town trips, five patients (20.8%) used oxygen, but most avoided traveling long distances. 

Oxygen usage during different modes of transportation

The oxygen usage during transport is demonstrated in Table 3.

**Table 3:** Oxygen usage during transport N/A: not applicable

Transport mode	Using oxygen	Helpful	Not using oxygen	Not performing activity
Driving a car	0	N/A	2 (8.3%)	22 (91.7%)
Traveling by road (bus/taxi)	2 (8.3%)	2 (100%)	4 (16.7%)	18 (75%)
Traveling using private vehicles	16 (66.7%)	16 (100%)	5 (20.8%)	3 (12.5%)
Traveling by railways	2 (8.3%)	2 (100%)	1 (4.2%)	21 (87.5%)
Traveling by airway	2 (8.3%)	2 (100%)	1 (4.2%)	21 (87.5%)

Grid of themes and representation from transcripts

The grid of themes and representation from transcripts is demonstrated in Table 4.

**Table 4:** Grid of themes and representation from the transcripts

Domain	Categories	Synthetic themes	Representation from transcripts - voice
Physical health and function	Positive perceptions	Benefit	“I breathe better with oxygen.”
		Symptom relief	“When I use oxygen, I feel less breathless and I don’t feel exhausted in little things.”
		Enabler	“Because of oxygen, despite of my bad lungs, I was able to go out with my whole family last year.”
		Keeping alive	“Without oxygen, I guess I will not be able to breathe.”
		Faith in doctor	“If it would be harmful, why would doctors prescribe me oxygen”
	Negative perceptions	Restriction	“I can’t go anywhere out for long due to this oxygen” “I can’t get to be moving around freely. I have to think twice before going anywhere, pack everything and carry it along.”
		Declining disease status	“My condition is getting worse. Oxygen is also not helping me much”
	Mixed	Adaptation	“Without oxygen, as I start to feel breathless, I sit for 5-10 minutes and then again continue.”
		Compromise	“My oxygen level drops and I don’t have any other option. So I have made up my mind that I have to use oxygen all life.”
Psychological	Positive	Feels good	“….feels relaxed with oxygen”
		Sense of security	“Whenever my symptoms worsen, I take oxygen and I am better.”
		Confidence	“Oxygen gives me confidence in doing my work by myself”
	Negative	Fear of dependency	“But I feel if I use it all the time, then I will get addicted to it and then I will never get better without oxygen. “
		Embarrassment	“It makes me feel as I have some disability and I don’t wish to be looked at like that. “
		Feeling of burden	“I feel I am not able to actually do anything for my family and they have to do all things”
		Uncertainty	“But I am not sure that how long this will help me. My condition is not getting any better.”
		Disappointment	“I am using this oxygen from so long now, still it doesn’t get any better”.
		Frustration	“I feel oxygen is just a pain.” “This is a big headache.”
		Hopelessness	“I am tired. I don’t have the will to live now.” “It’s better if I die.”
	Mixed	Acceptance	“Now, I have made up my mind that oxygen is my necessity and I have to use it.”
		Coping	“But now as we started accepting this reality, with all equipment’s, doctor’s and family support, I am able to do many things.”
		Knowledge and understanding	“I don’t think it is harmful, but I really don’t know how much it is effective also.” “I wonder sometimes does using oxygen continuously has any side effects. So I try to minimize my oxygen use as I feel too much oxygen is not good.”
Social	Positive	Family Support	“My family has always supported me and never tried to belittle me.” “Everybody in the family is worried about my health and wants me to recover.”
	Negative	Social isolation	“Now, I and my wife are just stuck at home as I cannot travel without oxygen.” “I don’t usually eat with everyone like before. “
		Attitude of others	“…as I don’t like people looking at me like that.”
		Restricted participation	“But as I had to start taking oxygen, I stopped taking tuitions as I used to feel breathless on talking for longer periods of time.” “Earlier, I used to attend all religious and my family functions. But now, I don’t.”
Environment	Positive	Support	“Everybody knows in the society and also seeing other people in my rehabilitation center gave me confidence.”
	Negative	Financial burden	“I don’t know how we are going to manage so much financially with my equipment, and my wife’s medicines.”
		Mobility curtailment	“I used to be out all day for work and then roam around in society but now everything is stopped and I have nothing to do.” “And now with the pandemic thing, it has added more. Like already we were in four walls and now I feel all the more caged.”

QOL

For the domains, consisting of physical health, psychological, social relationships, and environment, the mean values were found to be 48.33 ± 10.66, 54.79 ± 13.7, 55.75 ± 11.1, and 60.25 ± 12.6, respectively, with a standard error of the mean of 2.17, 2.79, 2.26, and 2.59, respectively; a 95% confidence interval for the mean consisting of the lower bound and upper bound of 43.828 and 52.83, 49.01 and 60.57, 51.06 and 60.44, and 54.89 and 65.61, respectively; and a range of 25 to 69, 31 to 81, 31 to 75, and 38 to 75, respectively.

## Discussion

This study critically evaluates the perceptions and impacts of LTOT on functioning, participation, and QOL among patients with chronic respiratory diseases in India, exploring a gap in the literature by addressing both the clinical outcomes and sociocultural dynamics surrounding its use through a mixed-method approach. While the prescription of LTOT focuses on survival benefits and managing symptoms, its acceptance and adherence remain low [13] due to several factors.

The adoption of LTOT among the participants of the present study varied widely, with a notable discrepancy between prescribed full-time use and actual usage patterns. Earnest et al. described a series of three stages consisting of initiation of oxygen use, negotiation, and compromise with full-time oxygen use while explaining different patterns of adherence to LTOT in their path toward full-time oxygen usage [13]. A significant number of participants used oxygen only part-time, citing physical discomfort and the stigmatization associated with visible medical equipment as major deterrents. These findings align with Steiner JF's notion that understanding patient behavior requires delving into the "why" behind actions, not just the "what" [15]. The cultural stigma attached to visible signs of illness, combined with the physical encumbrance of LTOT, and lack of specific instructions regarding oxygen usage complicates patient adherence, echoing the dissonance noted by Arnold et al. between health professional beliefs and patient realities [16]. The patients' faith and trust in physicians, associated with higher compliance [13,17], were similar to the findings of the present study.

In the Indian context, health and medicine are often viewed through a holistic lens, significantly shaping patient attitudes toward technologically dependent treatments like LTOT. Many patients perceive the mechanical nature of LTOT as a deviation from natural healing practices, leading to reluctance and sporadic adherence. This cultural hesitation is in contrast with Western settings, where there is a greater acceptance of medical technologies, influenced by a cultural trust in scientific progress and innovation. Bridging this cultural gap requires healthcare providers to integrate an understanding of these traditional beliefs with modern medical practices, potentially improving LTOT acceptance and adherence through a sensitive and informed approach to patient education that respects these cultural nuances [17,18].

Despite the prescription, individuals described making self-decisions on the basis of their perceived need for oxygen during activity and monitoring their symptoms, such as dyspnea and oxygen saturation, for decision-making of LTOT. The patient frequently believes that oxygen is prescribed to treat dyspnea [19]. While oxygen therapy does not entirely alleviate symptoms, it is thought to prevent their worsening. In addition, some patients adopt strategies such as increasing the oxygen flow during activities, pacing their actions, avoiding excessive strain, and using longer tubing to manage their condition better. Previous research has highlighted themes of adaptation, like mixed blessings, compromise, and trade-offs associated with the challenges of using oxygen. These studies reveal that patients often adopt a resigned acceptance, enduring the inconveniences of continuing their treatment with LTOT [9,19,20,21].

In the present study, the majority of the patients responded that with oxygen, they are at least able to perform their daily activities. Around 30% of the participants required assistance during ADLs like bathing and changing clothes as they require a high amount of energy. LTOT has been shown to enhance patients' ability to perform daily activities and manage symptoms like dyspnea, better sleep, and reduced fatigue, paralleling findings from previous studies that report improved functional independence and QOL and increased capacity with consistent oxygen use [19,22,23].

Conversely, many patients experienced a worsening of their condition despite using oxygen therapy, leading to skepticism about its effectiveness. This decline can be linked to the progressive nature of chronic respiratory diseases like COPD and ILD, which inherently limit functionality [10,24,25]. Research has identified physical inactivity as a key predictor of mortality in these conditions [26]. Although LTOT reduces their mobility, most patients were able to navigate their homes independently with oxygen therapy. However, the physical constraints imposed by the equipment often conflict with the benefits, such as increased capacity and stamina, creating a tension highlighted by Earnest et al. [13]. Furthermore, Okubadejo noted that COPD patients using LTOT scored lower on the Nottingham Extended Activities of Daily Living scale compared to those not on LTOT [27].

Patients engaging in rehabilitation and structured exercises reported significant benefits from using oxygen, which improved their exercise capacity, and enhanced feelings of confidence and overall well-being. They used LTOT during these activities, even if not throughout the entire day. Some of the patients described that exercises with oxygen therapy helped them to be relatively independent and gave them a sense of well-being. These activities, facilitated by LTOT, helped patients manage their symptoms more effectively and maintain some level of independence. Patients who received education from healthcare personnel were less likely to report problems with oxygen [26]. This underscores the importance of pulmonary rehabilitation in enhancing the therapeutic outcomes of LTOT [26,27]. Supporting this, a study by Sahin et al. showed improvements in six-minute walk distances and dyspnea scores in LTOT patients undergoing pulmonary rehabilitation [28]. It has been postulated that physical activity contributes to less fatigue and dyspnoea, with concomitant improvement in mental health and social engagement, resulting in improved QOL [16].

While LTOT helps many patients maintain mobility within their homes without assistance, the cumbersome nature of the equipment often restricts their overall physical activity as supported by other studies [1,9,16,29,30]. Problems include logistical and operational challenges of managing and adjusting heavy, oxygen systems, which can lead to feelings of being "stuck" reducing patients' QOL. In addition, the requirement for frequent adjustments and the fear of equipment failure discourage active and social lifestyles. Some patients choose to use oxygen post-exertion, using it as a relief measure rather than a constant aid. According to Mussa et al., in oxygen-dependent patients, perceived mobility is highly impacted by satisfaction with an LTOT device [1]. 

In Indian culture, strength, self-sufficiency, and active community involvement are highly valued and the dependency signified by LTOT created a perception of stark dissonance. Patients often perceive themselves as burdens, which exacerbates guilt and adversely affects their mental health and overall QOL. This internal conflict between needing medical support and fulfilling culturally assigned roles contrasts sharply with Western norms, where health management is viewed more as a personal responsibility, and using medical aids like oxygen therapy is considered empowering rather than a sign of weakness [18].

In addition, it carries stigmatization in society [13], because of which the patients described feelings of frustration, disappointment, embarrassment, dependency, guilt, shame, hopelessness, and restriction with oxygen therapy [8,9,13,19,24,31,32]. The requirement to adhere to specific oxygen flow rates and usage schedules also imposes constraints that can interfere with work, social engagements, and leisure activities. According to Arnold et al., to overcome the feelings of embarrassment and physical barriers to usage of their ambulatory oxygen equipment, social support was essential [16]. Adherence to oxygen consumption is promoted primarily by family and friends [13]. Western countries often have a more individualistic societal structure, prioritizing privacy and personal autonomy where the community typically plays a less significant role in daily life. In such settings, medical aids like oxygen therapy are generally viewed as part of personal health management, which diminishes the stigma linked to medical dependency and allows patients to use their treatments more freely and openly, without fear of societal judgment [18].

In India, the economic impact of LTOT is particularly severe due to lower average incomes and inadequate health insurance coverage that strain patients and their families. Unlike many Western countries, where health insurance covers most costs related to chronic disease management, including equipment like oxygen concentrators, support for home-based care in India is minimal. This lack of support means that many patients are underinsured or uninsured, making the costs associated with oxygen therapy equipment daunting [33]. In addition, the financial pressures of managing a chronic illness can cause considerable psychological distress, negatively impacting the patient's QOL and well-being [17,33].

The thematic analysis of patient interviews revealed profound insights into psychological and social repercussions. This sentiment underscores the social withdrawal patients experience, not only from their own hesitance but also from perceived changes in the behavior of others toward them. The visibility of oxygen equipment often brands patients with a visible marker of illness. This quote underscores the social identity shift that patients experience, where the presence of medical equipment overshadows their identity.

Community participation, which is central to life, particularly in Indian culture, is significantly hampered. Patients report a decrease in attending social gatherings, which were once a vital part of their social life. The retreat from community activities not only isolates patients but can also lead to loneliness and depression, further deteriorating their QOL. The emotional burden of living with chronic respiratory diseases is compounded by the stigma associated with dependency on medical aids. Patients often feel they are a burden to their families, which affects their self-esteem and mental health. Such sentiments highlight the emotional strain and guilt that accompany the physical challenges of managing their condition.

Furthermore, the constant visibility of their condition through the use of LTOT can exacerbate feelings of vulnerability and anxiety [17]. The psychological impact of such visibility cannot be understated, as it affects how patients view themselves and interact with the world. The analysis also highlighted resilience and coping mechanisms that patients develop. Despite the challenges, some patients expressed a determination to maintain normalcy in their lives showing the adaptive strategies employed to manage their condition and maintain QOL.

Strengths and limitations

The study makes significant contributions to understanding the complex interplay of cultural, economic, and medical factors in the management of chronic respiratory diseases through LTOT. It lays a foundation for future research and policy interventions aimed at improving the effectiveness and acceptance of LTOT in culturally diverse populations. Perceptions of patients regarding oxygen therapy can be used as a guide in devising solutions for their problems and in turn improve adherence to oxygen to achieve clinical outcomes.

However, the study also has certain limitations that involved the sample size, which was sufficient for qualitative analysis but relatively small for generalizing the quantitative findings to the broader population of LTOT users in India. Second, the study relies on self-reported data due to which the validity of results may be compromised due to limitations inherent in this study design, such as recall bias, respondent’s ability to understand the questions, response bias, and differences in how respondents interpret the rating scales used in activity and participation checklist. The wide range in characteristics of oxygen usage, ability to do exercises, and QOL scores can be attributed to the diverse nature of participants.

## Conclusions

The study concluded that LTOT has been perceived to be a lifesaving intervention by a majority of the chronic respiratory disease patients of progressive severity and have experienced varied issues with oxygen therapy delivering themes, which in turn have affected their QOL. In addition, patients on LTOT strive to adapt to the usage of oxygen by negotiating its use in their basic activities of daily living and lifestyle with varied perceived benefits and shortcomings. Overall, a lack of awareness and knowledge regarding the purpose, dosage, benefit, and usage of oxygen therapy was evident and needs attention. The economic challenges associated with maintaining and using LTOT equipment hinder its accessibility and consistent use, thereby offering insights that could aid in policymaking. The cultural beliefs surrounding health and medicine in India also play a crucial role in shaping patients’ attitudes toward such medical interventions. For future research, it is essential to address the limitations noted in this study by incorporating longitudinal data to better understand the long-term implications of LTOT.

## Appendices

Semi-structured interview

1. Do you use your oxygen full-time or part-time? Is it as prescribed by the physician?

2. For which activities you do not use oxygen? What are the reasons why are you not using it?

3. Do you go out of your home? How frequently? How do you manage oxygen therapy while going out?

4. Do you go out of town for work or leisure? How do you manage oxygen therapy while going out?

5. Do you need help or assistance from a caregiver or walking aid to move inside or outside the house?

6. Are you able to do exercise with oxygen therapy? What is your thought on doing exercise as prescribed with oxygen therapy?

7. How do you think oxygen therapy affects in ability to do exercise?

8. Do you feel oxygen has relieved your symptoms? Which symptoms are relieved? Can you list them?

9. Do you feel oxygen is harmful to your body in any way?

10. How do you think oxygen therapy has helped you?

11. What problems do you face with the use of oxygen therapy? How do you cope with these problems?

12. Do you feel being on oxygen therapy has affected your role in your family or society? If yes, how?

13. Do you have enough information or help with respect to oxygen therapy equipment?

14. Do you have any worries about living with oxygen therapy? If yes can you explain what are they?
